# Nitric Oxide Is Involved in Heavy Ion-Induced Non-Targeted Effects in Human Fibroblasts

**DOI:** 10.3390/ijms20184327

**Published:** 2019-09-04

**Authors:** Megumi Hada, Premkumar B. Saganti, Francis A. Cucinotta

**Affiliations:** 1 Chancellor’s Research Initiative, Radiation Institute for Science & Engineering, Prairie View A&M University, Prairie View, TX 77446, USA; 2 Department of Health Physics and Diagnostic Sciences, University of Nevada Las Vegas, Las Vegas, NV 89154, USA

**Keywords:** ionizing radiation, chromosome aberrations, non-targeted effects

## Abstract

Previously, we investigated the dose response for chromosomal aberration (CA) for exposures corresponding to less than one particle traversal per cell nucleus by high energy and charge (HZE) particles, and showed that the dose responses for simple exchanges for human fibroblast irradiated under confluent culture conditions were best fit by non-linear models motivated by a non-targeted effect (NTE). Our results suggested that the simple exchanges in normal human fibroblasts have an important NTE contribution at low particle fluence. Nitric oxide (NO) has been reported as a candidate for intercellular signaling for NTE in many studies. In order to estimate the contribution of NTE components in induced CA, we measured CA with and without an NO scavenger in normal skin fibroblasts cells after exposure to 600 MeV/u and 1 GeV/u ^56^Fe ions, less than one direct particle traversal per cell nucleus. Yields of CA were significantly lower in fibroblasts exposed to the NO scavenger compared to controls, suggesting involvement of NO in cell signaling for induction of CA. Media transferred from irradiated cells induced CA in non-irradiated cells, and this effect was abrogated with NO scavengers. Our results strongly support the importance of NTE contributions in the formation of CA at low-particle fluence in fibroblasts.

## 1. Introduction

Long-duration space flight in deep space is becoming a closer reality. Radiation in deep space is the most significant health concern. Space radiation consists of a combination of energetic particles of varying masses and energies called galactic cosmic rays (GCR). Understanding the dependence of the induction of genomic instability on the linear energy transfer (LET) and the dose of the charged particles is critical in order to accurately assess the cancer risks from exposures during long-term space missions to the Moon or Mars [1,2]. Radiation quality, including increased tumor malignancy for high LET radiation, non-targeted effects (NTE), and dose-rate effects are among the largest uncertainties that affect predictions of space radiation induced cancer risk. Current cancer risk projections for cosmic ray proton and HZE particle exposures are extrapolated from high dose γ-rays derived from human epidemiology data using quality factors and the dose and dose-rate reduction effectiveness factor (DDREF), assuming a linear no-threshold dose response. However, experimental data for risks at low doses of radiation are highly uncertain and biological responses to low doses, in the range received by astronauts during a space mission, may be affected by non-DNA targeted effects that could produce a non-linear response.

The yield of CA has been shown to increase in the lymphocytes of astronauts after long-duration missions of several months in space [3]. Chromosome exchanges, especially translocations, are positively correlated with many cancers, and are therefore a potential biomarker of cancer risk associated with radiation exposure [4,5,6,7]. In addition, relative biological effectiveness (RBE) factors for chromosomal aberrations are similar to RBEs observed for induction of solid tumors in mice [4,6,7]. Therefore, CA may continue to remain as a useful biomarker for cancer risks and for comparisons with other biomarkers in the absence of human data for galactic cosmic rays [1,8].

Recently, we reported that the dose response for induction of CA is not linear in human fibroblasts after HZE nuclei exposures of less than one particle traversal per cell nucleus [9]. The dose response for CA was best fit with a nonlinear model motivated by a non-targeted effect (NTE) due to aberrant cell signaling [7,10]. Our results suggest that NTE is an important contributor to chromosome exchanges in normal human fibroblasts irradiated with low particle fluence. We have also found additional evidence for NTE in low dose experiments measuring γH2AX foci, a marker of double strand breaks (DSB), and split-dose experiments with human fibroblasts [9]. Since the assumption of a linear dose response for biological effects of high-LET radiation is fundamental in radiation protection methodologies, our recent findings could have a major impact on current risk assessment predictions for astronauts.

In this study, we tested induction of CA with media transfer using Fe-ion exposure at Brookhaven National Laboratory (BNL), Upton NY, in order to obtain more direct evidence of NTE involvement in our experimental protocols to assess our earlier model calculations. Medium from irradiated cells was found to increase frequency of CA in non-irradiated cells.

Several mechanisms involved in non-targeted effects have been reported for many different endpoints [11]. Direct inter cellular communication via Gap junction in signaling the response from an irradiated cell to non-irradiated cell has been shown by studies with α particles, β particle, γ-rays and HZE radiations [12,13]. Secreted diffusible factors such as TGF- β, interleukin-8, serotonin, ROS, cytokines, calcium ions and small RNA have been also reported to induce NTE [14,15,16].

With preliminary screening of signaling involved in our system, the Gap junction inhibitor and reactive oxygen species (ROS) scavenger did not show a significant effect in yield of CA whereas the NO scavenger showed a significant effect. In order to estimate the contribution of NTE components in CA induced by radiation, we studied the dose response of CA frequency with and without the NO scavenger.

## 2. Results

**Figure 1:**
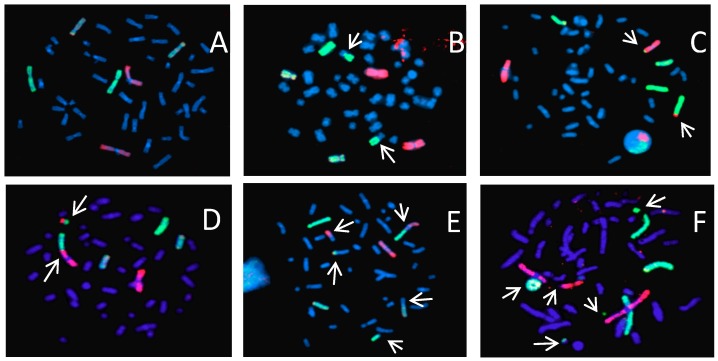
Examples of chromosome painting in human fibroblasts (82-6hTERT) with a 3 color whole chromosome FISH: Chromosome 1 (red), chromosome 2 (green), and chromosome 4 (yellow). Chromosome aberrations were identified by arrows as simple (reciprocal exchanges between two chromosomes) or complex-type exchanges (exchanges involving a minimum of 3 breaks in 2 or more chromosomes). **A**: normal; **B**: simple exchange between chromosome 2 and other chromosome; **C**: simple exchange between chromosome 1 and 2; **D**: simple exchange between chromosome 1 and 2 (dicentric); **E**: complex exchange in chromosome 1, 2 and other chromosome, simple chromosome in chromosome 4; **F**: Ring in chromosome 4, and breaks in chromosome 1 and 2.

Figure 1 shows images of 3-color chromosome FISH in which chromosome 1 (red), chromosome 2 (green), and chromosome 4 (yellow) are identified. All chromosomes are labeled by DAPI (4, 6-diamidino-2-phenylindole). Undamaged chromosomes are shown in Figure 1A. Other panels show chromosomes that are damaged in various ways (see figure legends). All types of detectable aberrations in chromosome 1, 2 and 4 were scored and whole genome equivalent frequencies of aberrations were calculated.

**Figure 2:**
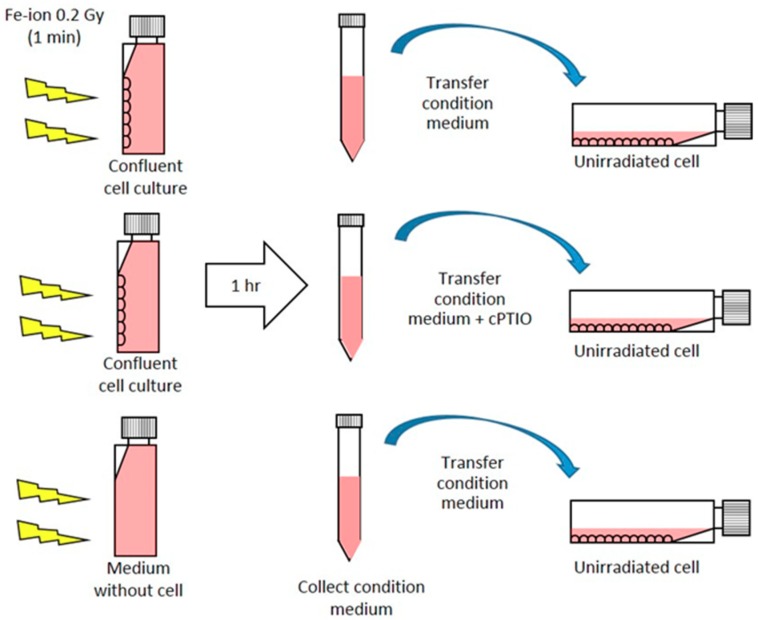
Scheme of media transfer experiment. Conditioned media exposed and collected from cell culture or medium only to 0.2 Gy Fe-ion were transferred to un-irradiated cells.

**Figure 3:**
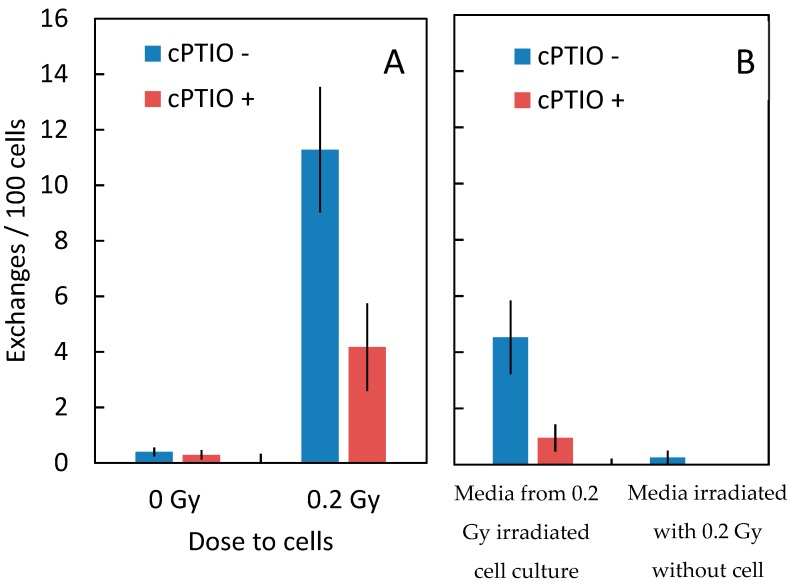
Graphical representation of the data shown in the Table 1. **A**: Frequencies of total chromosome exchanges in cells exposed to 0 Gy or 0.2 Gy Fe ion (1000 MeV/u) with and without cPTIO. **B**: Frequencies of total chromosome exchanges induced in non-irradiated cells by transferred conditioned media. Media from cell culture irradiated with 0.2 Gy Fe-ions (1000 MeV/u) on the left and media irradiated with Fe-ion without cells on the right. Error bars indicate the standard error of the mean values.

To test involvement of NTE in CA, media were taken from 0.2 Gy Fe-ion exposed culture at 1 hr after exposure and transferred to non-irradiated cells (Figure 2 and Figure 3, Table 1).

Non-irradiated cells show an increased frequency of chromosome aberrations with medium transfer. When nitric oxide scavenger, cPTIO, was added to the transfer medium, chromosome aberration was not increased, suggesting that nitric oxide in irradiated medium was involved in inducing chromosome aberrations in non-irradiated cells. Maeda et al. reported that irradiated medium can increase sister chromatid exchange in Chinese hamster ovary cells [17]. In our experiment, medium irradiated with 0.2 Gy Fe-ions without cells did not induce chromosome aberrations in non-irradiated cells. These results suggest that CA induced in non-irradiated cells by media transfer were due to NO released by irradiated cells into the medium.

We also tested gap junction inhibitor (18αGA), ROS scavenger (1% DMSO), and long lived radicals scavenger (ASC2-P) in order to screen signaling involved in our system. Addition of 18αGA, DMSO and ASC2-P to cell culture did not show a significant effect on the yield of CA (Supplementary Table S1).

**Figure 4:**
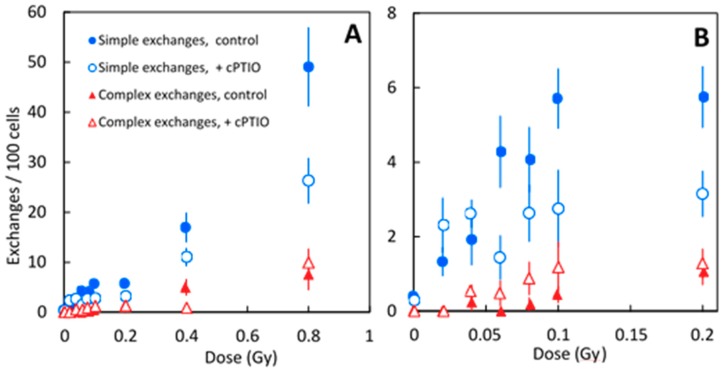
Graphical representation of the data shown in the Table 2. Frequencies of simple and complex types of chromosome exchanges exposed to Fe-ions (600 MeV/u) with or without the NO-scavenger cPTIO. **A**: Results across all doses. **B**: Results at lower doses. Error bars indicate the standard error of the mean values.

To quantify the extent of the CA induced by NTE, dose responses to Fe (600 MeV/u) exposure were obtained with and without cPTIO (Table 2, Figure 4). No increases occurred in the frequency of simple exchange in the dose range 0.06–0.2 Gy (less than 1 hut per nucleus), as we shown in a previous report [9].

When the exposure dose was higher than 0.06 Gy, cPTIO reduced 35–50% of simple exchanges induced by 600 MeV/u Fe-ions, but not complex exchanges, suggesting NTE-induced CA were only simple type exchanges. When the exposure dose was lower than 0.04 Gy, the difference in CA obtained with or without cPTIO was not statistically significant. NO released in the medium induced at least 1/3-half of simple exchanges observed in fibroblasts exposed to Fe-ions. These results support our previous findings from modeling [7,9,10].

## 3. Discussion

Interaction between hit and non-hit cells after exposure to ionizing radiation have been known for many years, and a variety of endpoints has been reported [11,18]. It is well known that ionizing radiation can stimulate the release of signaling molecules into the extracellular space or through gap junctions [19,20]. Although several mechanisms of how this signaling process induces a radiation-like effect have been proposed, this process is not fully understood.

In order to screen for the signaling involved in our system, gap junction inhibitor (18αGA), NO scavenger (cPTIO), ROS scavenger (1% DMSO), and a long-lived radical scavenger (ASC2-P) were added to cell culture and CA was measured. 18αGA, DMSO and ASC2-P did not show a significant effect on the yield of CA (data not shown), whereas the NO scavenger showed a significant effect. In our experimental system, conditioned media from irradiated cells induced CA in non-irradiated cells and this effect was abrogated by addition of cPTIO (Figure 2), indicating that NO released from irradiated cells into the media induced CA.

The dose response of CA frequency for 600 MeV/n Fe with and without cPTIO shows that yields of CA were significantly lower in fibroblasts exposed to the NO scavenger compared to controls (Figure 3). This indicates that at least half of the CA induced by Fe ion exposure occurred through NTE involved with NO released from irradiated cells. These results support strongly our previous modeling studies [7,9,10,21]. Matsuya et al. [22] predicts DNA damages in non-hit cells induced by NTE-involved signals have a lower repair efficiency compared to damages induced in hit cells. Further study is needed to understand the mechanisms of CA induction by NTE-induced DNA damage.

CA is the one of the in vitro surrogate endpoints and a well-established biomarker for cancer risk. Bonassi et al. reported that chromosome aberrations, especially translocations, are positively correlated with many cancers [4]. The yield of CA has been shown to increase in the lymphocytes of astronauts after long-duration missions of several months in space [3,23,24]. Ballarini et al. predicted, from a modeling study, cell death in human (and hamster) fibroblasts exposed to different radiation types including Fe ions, assuming that asymmetrical exchanges (dicentrics, rings and large deletions) induced clonogenic cell death [25]. Higher-than-linear chromosome aberration induction may not automatically imply a higher-than-linear cancer risk because many cells containing complex types of aberrations or asymmetrical exchanges are likely to die. In our study with low dose exposure, most simple exchanges observed were symmetrical exchanges, with potential for transmittal of these CA to progeny.

## 4. Materials and Methods

### 4.1. Cell Culture

We used hTERT-immortalized human normal skin fibroblast cells 82-6 [26], a kind gift from Dr. J. Campisi (Lawrence Berkeley National Laboratory, Berkeley, CA, USA); these cells were grown as monolayers at 37 °C under 5% CO_2_ in Dulbecco’s Modified Eagle Medium (GIBCO, Invitrogen, Carlsbad, CA, USA) supplemented with 10% fetal bovine serum, antibiotic-antimycotic (1x) and L-glutamine (2 mM). Cells were irradiated in a confluent state with accelerated heavy ions. For the test of the Gap junction inhibitor, 50 µM of 18 α-glycyrrhetinic acid (18α-GA, Sigma-Aldrich, St. Louis, MO, USA) was added to cell culture 30 min before the irradiation. For the test of the ROS scavenger, 1% of dimethylsulfoxide (DMSO, Sigma-Aldrich, St. Louis, MO, USA) was added to the cell culture 30 min before the irradiation. For the test of the long-lived radical scavenger, 1 mM of l-ascorbic acid 2-phosphate (ASC2-P, Sigma-Aldrich, St. Louis, MO, USA) was added to the cell culture just after irradiation. For the test of the NO scavenger, 3 µM of 2-(4-Carboxyphenyl)-4,4,5,5-tetramethylimidazoline-1-oxyl-3-oxide potassium salt (cPTIO, Sigma-Aldrich, St. Louis, MO, USA) was added to the cell culture 2 h prior to irradiation.

### 4.2. Irradiation

Cells were exposed to 600 MeV/u (LET 175 keV/µm) or 1000 MeV/u (LET 151 keV/µm) Fe ions, or 170 MeV/u (LET 99 keV/µm) Si ions at the NASA Space Radiation Laboratory (NSRL) of the Brookhaven National Laboratory (BNL) [27]. Doses ranged from 0.02 Gy to 0.8 Gy. Dose rates were changed between 0.02 and 0.8 Gy /min, varying depending on the dose delivered, with each exposure being 1 min. Mean hits per cell were obtained based on the particle LET and area of the cell nucleus of 82-6 cells (162 µm^2^) [9]. Exposed doses were selected as 0.1–4.6 hits per cell nucleus following our previous study on modeling [9]; 0.2 Gy Fe (600 MeV/u) is estimated about 1 hit per cell nucleus.

### 4.3. Media Transfer

Following irradiation, the cells were left undisturbed until media transfer, which was performed one hour after irradiation. The cell cultures from 0.2 Gy exposed flasks were transferred to 15 mL centrifuge tubes and centrifuged at 370× *g* for 5 min. The supernatant was then filtered through 0.22 µm polyethylsulfonate syringe filter. The media from irradiated cells contain factors secreted by the irradiated cells considered as conditioned media. The media from non-irradiated cells were gently removed by aspiration and replaced with conditioned media.

### 4.4. Premature Chromosome Condensation (PCC)

The PCC technique was used to collect G2/M-phase chromosomes as previously described [9,28,29]. After irradiation, cells were allowed to repair for 16 h, and were then sub-cultured at low cell population density. After 10 h incubation, cells were arrested in mitosis by adding colcemid to a final concentration of 0.1 μg/mL in the culture media, and then cells were incubated for an additional 6 h. Approximately 30 min before collection, 50 nM of calyculin A (Wako Pure Chemical Industries Ltd., Osaka, Japan) was added to the culture media to condense the chromosomes in the G2 phase of the cell cycle.

### 4.5. Fluorescence In Situ Hybridization (FISH)

Chromosome spreads were prepared as described in [30] and were hybridized in situ with a combination of three fluorescence whole chromosome human DNA probes for chromosomes 1 (red), 2 (green) and 4 (yellow) (Aquarius, Cytocell, Oxford Gene Technology, Oxfordshire, UK), using the protocol recommended by the manufacturer. All chromosomes were then stained with DAPI. Chromosomes were analyzed with the Leica Cytovision fluorescence in situ hybridization (FISH) system which includes Leica fluorescent microscope with a charge-coupled device (CCD) camera and karyotyping software. Images of all cells with damaged chromosomes 1, 2 and 4 were captured electronically.

### 4.6. Classification of Chromosome Aberrations

Two bicolor chromosomes each containing a centromere were classified as an apparent reciprocal translocation and recorded as a single exchange event. Complex exchanges were scored when it was determined that an exchange involved a minimum of three breaks in two or more chromosomes [28]. An exchange was defined as “simple” if two breaks in two chromosomes were noted, that is, dicentrics and translocations. Incomplete translocations and incomplete dicentrics were included in the category of simple exchanges, assuming that in most cases the reciprocal fragments were below the level of detection. Each type of exchange (dicentrics, apparently simple reciprocal exchanges, incompletes or complex) was counted as one exchange, and values for total exchanges were derived by adding the yields. When two or more painted chromosomes were damaged, each was scored separately. Since uncolored chromosomes cannot be identified, there is a potential for under-estimation of complex exchanges in this method. Although there are limitations with the 3-color FISH technique, this method is useful for analyzing a large amount of chromosomes efficiently. At least 1000 chromosomes (or reached 50 aberrant spreads) per sample were analyzed.

### 4.7. Statistical Analysis

The frequencies of chromosomal aberrations in painted chromosomes were evaluated as the ratio between aberrations scored and total cells analyzed. Several studies have indicated that the distribution of radiation damage among chromosomes is random, and the yield of exchanges measured within the first division after exposure is proportional to the DNA content of the chromosome analyzed, with some fluctuation of data [31]. Therefore, the frequencies of exchanges in individual chromosomes can be extrapolated to whole-genome equivalents using a modified version of the Lucas et al. [32] formula, Fp = 2.05[fp(1 − fp) + fp1fp2 + fp1fp3 + fp2fp3]FG. Fp is the combined frequency of exchanges in all painted chromosomes, fp is the fraction of the whole genome comprised of the painted chromosomes, fp1, fp2, and fp3 are the fractions of the genome for each individual chromosome, and FG is the whole-genome aberration frequency. Using this formula, genomic frequency for 83-6hTERT fibroblasts was estimated as 2.48 times that detected in chromosome 1, 2, and 4.

Standard errors for aberration frequencies were calculated assuming Poisson statistics. Error bars in the figure represent the standard error of the mean values.

## 5. Conclusions

Nitric oxide released to the medium is involved in non-targeted effects in human normal fibroblasts exposed to Fe ions. Our results suggest the importance of NTE contributions in the formation of CA at low-particle fluence. The number of simple exchanges at low doses in fibroblasts will be greatly under-estimated without the NTE contribution. Our current and previous experimental studies provide important evidence that contradicts the linear dose response assumption used in radiation protection for HZE particles and other high-LET radiation at low doses. Although in vitro evidence cannot be automatically extrapolated to in vivo exposure scenarios like those experienced by astronauts exposed to GCR because GCR exposures occur at low doses and dose-rates, this is an important finding that should lead to other investigations in different cell types and comparison to other surrogate endpoints and model systems. It will be important to demonstrate NTE in tissue system irradiation at doses corresponding to less than one particle track per cell nucleus to strengthen our finding in cell culture models.

## Figures and Tables

**Table 1:** (**a**) Whole genome equivalent for frequency of CA per 100 cells in fibroblasts of 0.2 Gy Fe-ion (1000 MeV/u) exposure with and without the NO scavenger, 2-(4-Carboxyphenyl)-4,4,5,5-tetramethylimidazoline-1-oxyl-3-oxide potassium salt (cPTIO) added to the medium. (**b**) Whole genome equivalent for frequency of CA per 100 cells with medium transfer with and without the NO scavenger cPTIO.

Dose (Gy)	Treatment	Cells Scored (#)	Frequency of Chromosome Aberrations (%)		
			Simple	Complex	Total
(**a**)					
0	control	3701	0.40 ± 0.16	0	0.40 ± 0.16
0	cPTIO	2546	0.29 ± 0.19	0	0.29 ± 0.19
0.2	control	549	10.83 ± 2.21	0.45 ± 0.45	11.28 ± 2.26
0.2	cPTIO	416	4.17 ± 1.58	0	4.17 ± 1.58
(**b**)					
0	control	3701	0.40 ± 0.16	0	0.40 ± 0.16
0	Media transferred ^1^	657	3.77 ± 1.19	0.75 ± 0.53	4.53 ± 1.31
0	Media transferred + Cptio ^2^	1038	0.72 ± 0.41	0.24 ± 0.24	0.95 ± 0.48
0	Media transferred ^3^	1003	0.25 ± 0.25	0	0.25 ± 0.25

^1^ Media from 0.2 Gy Fe-ion irradiated cell culture were taken 1 hr after irradiation, filtered and transferred to non-irradiated cells. ^2^ Media from 0.2 Gy Fe-ion irradiated cell culture were taken 1 hr after irradiation, filtered, cPTIO was added, followed by transfer to non-irradiated cells. ^3^ Media without cells were irradiated with 0.2 Gy Fe-ions and transferred to non-irradiated cells.

**Table 2:** Whole genome equivalent for frequency of CA per 100 cells exposed to Fe ions (600 MeV/u) with and without cPTIO.

Dose (Gy)	Hit Per Cell	Treatment	Cell Score’D	Frequency of Chromosome Aberrations		
				Simple	Complex	Total
0	0	control	3701	0.40 ± 0.16	0	0.40 ± 0.16
0	0	cPTIO	2546	0.29 ± 0.17	0	0.29 ± 0.17
0.02	0.12	control	2236	1.33 ± 0.38	0	1.33 ± 0.38
0.02	0.12	cPTIO	1075	2.31 ± 0.73	0	2.31 ± 0.73
0.04	0.23	control	1030	1.92 ± 0.68	0.24 ± 0.24	2.17 ± 0.72
0.04	0.23	cPTIO	4630	2.62 ± 0.37	0.54 ± 0.17	3.16 ± 0.41
0.06	0.35	control	1157	4.28 ± 0.96	0	5.78 ± 1.11
0.06	0.35	cPTIO	1035	1.44 ± 0.59	0.48 ± 0.34	1.92 ± 0.68
0.08	0.47	control	1341	4.07 ± 0.87	0.18 ± 0.18	4.25 ± 0.89
0.08	0.47	cPTIO	1130	2.63 ± 0.76	0.88 ± 0.44	3.51 ± 0.88
0.10	0.59	control	2212	5.71 ± 0.80	0.44 ± 0.22	6.16 ± 0.83
0.10	0.59	cPTIO	630	2.75 ± 1.04	1.18 ± 0.68	3.93 ± 1.24
0.20	1.17	control	2112	5.75 ± 0.82	1.06 ± 0.35	6.81 ± 0.89
0.20	1.17	cPTIO	2127	3.15 ± 0.61	1.28 ± 0.39	4.43 ± 0.72
0.40	2.34	control	497	16.95 ± 2.91	4.99 ± 1.58	21.94 ± 3.31
0.40	2.34	cPTIO	875	11.04 ± 1.77	0.85 ± 0.49	11.89 ± 1.84
0.80	4.69	control	197	49.06 ± 7.86	7.55 ± 3.08	56.60 ± 8.44
0.80	4.69	cPTIO	325	26.69 ± 4.51	9.91 ± 2.75	36.60 ± 5.28

## Supplementary Materials

Supplementary materials can be found at https://www.mdpi.com/1422-0067/20/18/4327/s1.

## Abbreviations

18α-GA	18α-glycyrrhetinic acid
ASC2-P	L-ascorbic acid-2-phosphate
BNL	Brookhaven National Laboratory
CA	Chromosome aberrations
CCD	Charge-coupled device
cPTIO	2-(4-Carboxyphenyl)-4,4,5,5-tetramethylimidazoline-1-oxyl-3-oxide potassium salt
DAPI	4, 6-diamidino-2-phenylindole
DDREF	Dose and dose-rate reduction effectiveness factor
DMSO	Dimethylsulfoxide
DSB	Double strand breaks
FISH	Fluorescence in situ hybridization
GCR	Galactic cosmic rays
HZE	High atomic number and energy
LET	Linear Energy Transfer
MeV/n	Mega electron Volt per nucleon
NSRL	NASA Space Radiation Laboratory
NO	Nitric Oxide
NTE	Non-targeted effect
PCC	Premature Chromosome Condensation
ROS	Reactive Oxygen Species
SE	Standard Error
TAMU	Texas A&M University
