# Supplementary material for: Nitric Oxide Is Involved in Heavy Ion-Induced Non-Targeted Effects in Human Fibroblasts
Source: Int J Mol Sci. 2019 Sep 4;20(18):4327. doi: 10.3390/ijms20184327 (PMC6769611; doi:10.3390/ijms20184327)
Supplement: Supplementary file 1 [file ijms-20-04327-s001.pdf]

Table S1. Whole genome equivalent for frequency of CA per 100 cells exposed to Si-ions (170 MeV/u) or Fe ions (600 MeV/u) with and without addition of scavenger/inhibitor.

Exposure (Gy)	Hit per cell	Treatment	Cell scored	Frequency of chromosome aberrations		
				Simple	Complex	Total
Si ions (0.2 Gy)	2.05	control	1025	5.08 ± 1.11	0.48 ± 0.34	5.56 ± 1.16
	2.05	DMSO	1017	4.87 ± 1.09	0.49 ± 0.34	5.36 ± 1.14
	2.05	ASC2-P	1013	5.63 ± 1.17	0.24 ± 0.24	5.87 ± 1.20
Fe ions (0.2 Gy)	1.17	control	1487	3.83 ± 0.80	0.33 ± 0.24	4.17 ± 0.83
	1.17	18αGA	887	3.07 ± 0.93	0.84 ± 0.48	3.91 ± 1.05
